# Chromosomer: a reference-based genome arrangement tool for producing draft chromosome sequences

**DOI:** 10.1186/s13742-016-0141-6

**Published:** 2016-08-22

**Authors:** Gaik Tamazian, Pavel Dobrynin, Ksenia Krasheninnikova, Aleksey Komissarov, Klaus-Peter Koepfli, Stephen J. O’Brien

**Affiliations:** 1 Theodosius Dobzhansky Center for Genome Bioinformatics, St. Petersburg State University, Sredniy Prospekt 41A, St. Petersburg, 199004 Russia; 2 National Zoology Park, Smithsonian Conservation Biology Institute, 3001 Connecticut Avenue NW, Washington, 20008 D.C. USA; 3 Oceanographic Center, Nova Southeastern University, 8000 N. Ocean Drive, Ft. Lauderdave, 33004 Florida USA

**Keywords:** Reference-assisted assembly, Chromosome assembly, Alignment

## Abstract

**Background:**

As the number of sequenced genomes rapidly increases, chromosome assembly is becoming an even more crucial step of any genome study. Since *de novo* chromosome assemblies are confounded by repeat-mediated artifacts, reference-assisted assemblies that use comparative inference have become widely used, prompting the development of several reference-assisted assembly programs for prokaryotic and eukaryotic genomes.

**Findings:**

We developed Chromosomer – a reference-based genome arrangement tool, which rapidly builds chromosomes from genome contigs or scaffolds using their alignments to a reference genome of a closely related species. Chromosomer does not require mate-pair libraries and it offers a number of auxiliary tools that implement common operations accompanying the genome assembly process.

**Conclusions:**

Despite implementing a straightforward alignment-based approach, Chromosomer is a useful tool for genomic analysis of species without chromosome maps. Putative chromosome assemblies by Chromosomer can be used in comparative genomic analysis, genomic variation assessment, potential linkage group inference and other kinds of analysis involving contig or scaffold mapping to a high-quality assembly.

**Electronic supplementary material:**

The online version of this article (doi:10.1186/s13742-016-0141-6) contains supplementary material, which is available to authorized users.

## Background

Chromosome assembly is an important part of virtually any eukaryotic genome project. The number of assembled genomes increases each year and many of them are anchored to physical chromosome maps [1]. A robust *de novo* chromosome assembly requires not only mate-pair reads with different insert sizes, but also physical and genetic maps [2–4]. The large number of high quality assembled ‘reference genomes’ leads to an alternative approach – a reference-assisted chromosome assembly. Using this approach, the benefits of assembled chromosomes can be exploited without additional sequencing or map construction. These benefits include a known number of linkage groups and an estimated distance between markers, which is important for inferences of linkage and synteny. An assisted assembly also connects and orders large numbers of small contigs or scaffolds based on comparative analysis. In many cases, the initial number of contigs and scaffolds can exceed several hundred thousand following *de novo* assembly; working with such a fragmented genome can prove challenging [5]. Arranging contigs and scaffolds into putative chromosomes using information from the reference genome of a closely related species reduces the overall number of fragments from thousands to hundreds or dozens and also simplifies the annotation and analysis of different genomic features such as repeats, genes, single-nucleotide polymorphisms, copy number variations and segmental duplications.

A disadvantage of this approach is the introduction of occasional assembly errors driven by evolutionary chromosomal rearrangements. Even a closely related reference can differ in synteny from the target genome to some degree. The number of introduced assembly artifacts generally correlates with the evolutionary distance between the target and reference genomes [6] although rates of chromosome rearrangements are hardly clock-like, at least for mammals [7, 8]. These assembly artifacts are easily corrected if a physical map for the target genome is developed, using a tool such as the single molecule next-generation mapping system (Irys) developed by BioNano Genomics [9].

Multiple programs have been developed for reference-assisted chromosome assembly: Bambus [10], BACCardI [11], Projector2 [12], OSLay [13], ABACAS [14], MeDuSa [15], AlignGraph [16], Ragout [17], SyMap [18] and RACA [19]. Most of the listed tools were designed for bacterial or small genomes. For example, ABACAS is a convenient bacterial genome contiguation tool that may also be used for small eukaryotic genomes such as *Saccharomyces cerevisiae* (12.1 mega base pairs). However, ABACAS is not efficiently scaled to use with the large genomes typical of vertebrate species.

SyMap was designed to facilitate reference-assisted chromosome assembly for eukaryotic genomes; however, it has important limitations. SyMap uses MUMmer [20] or NUCmer [21] for the alignment phase, requires a separate structured query language (SQL) database to work efficiently and takes a very long time to align large genomes to each other.

The most promising approach for reference-assisted assembly is based on using several reference genomes instead of a single one. RACA implements such an approach, using alignments of target, reference and outgroup genomes as inputs to generate predicted chromosome fragments (PCFs) [19]. However, RACA also requires additional evidence from mate-pair libraries for joining genome fragments, while most *de novo* sequenced genomes have no such libraries available. Furthermore, RACA requires extensive computations for assembling chromosomes.

In this paper we introduce Chromosomer – an open-source cross-platform software that automates the reference-assisted building of genomic chromosomes and is especially effective for large genomes (> 1 giga base pairs). Chromosomer constructs draft chromosomes based only on alignments between fragments (contigs or scaffolds) to be arranged and a reference genome, thereby improving analytical and annotation opportunities for the index species assembly. Although Chromosomer does not use any sophisticated models or algorithms for chromosome assembly, we show that its results are comparable with state-of-the-art assemblies and can be used for further genomic analysis.

## Findings

### Algorithm

To map fragments to a reference genome, Chromosomer uses results of pairwise alignments between the fragments (contigs and scaffolds) and the chromosomes of the reference genome. The alignments are required to have associated score values that reflect the length and identity of the aligned regions (for example, the BLAST bit score [22]). In addition, the start and end positions of aligned regions in both the fragments and the reference chromosomes are required.

**Fig. 1:**
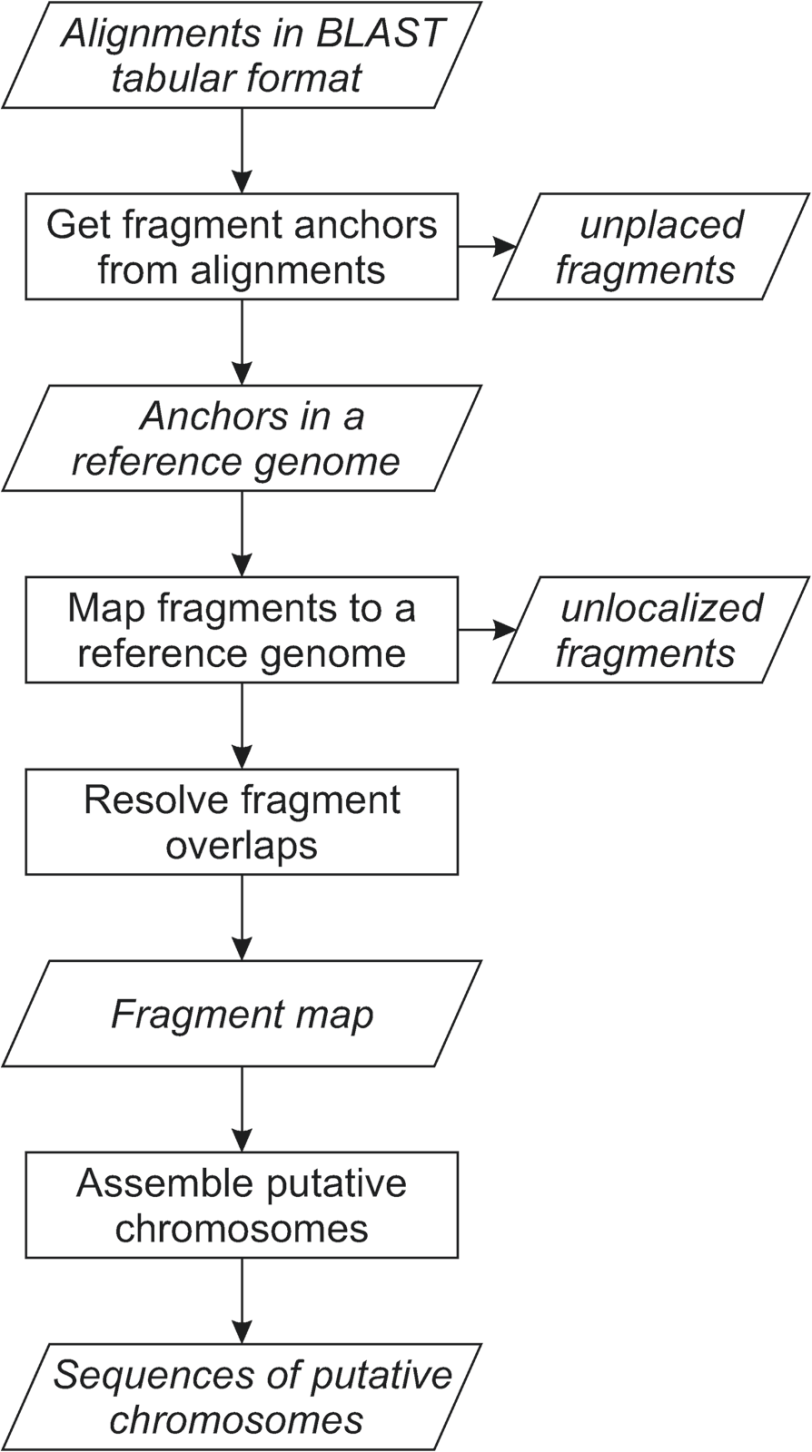
Chromosomer reference-assisted assembly workflow. Rectangles correspond to procedures applied to datasets, which are denoted in skewed rectangles

Chromosomer analyzes alignment positions and scores to map fragments to a reference. The mapping process takes the following steps (see Fig. 1).

**Fig. 2:**
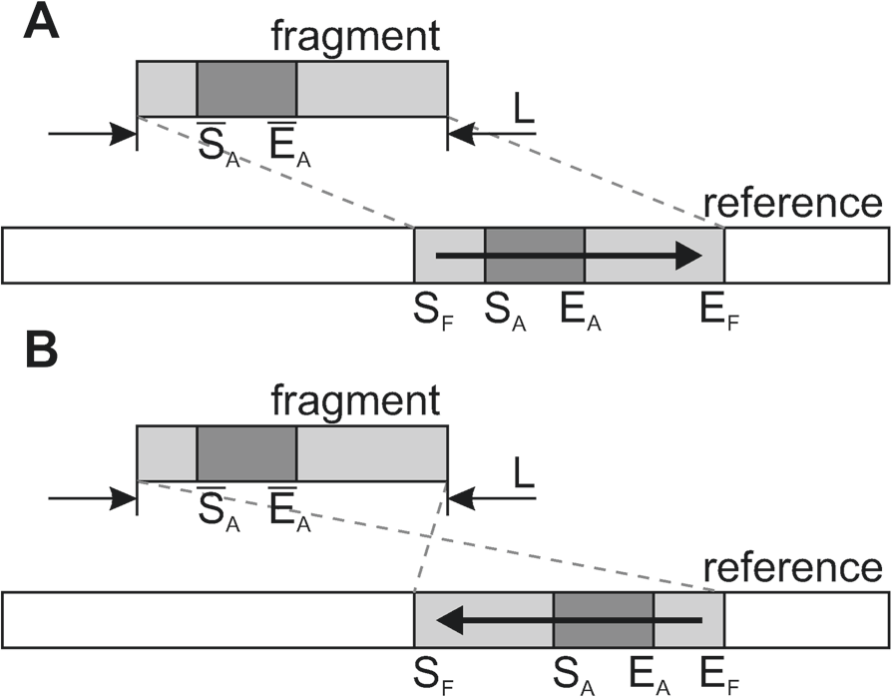
Alignment-based fragment-to-reference mapping. The alignment used for locating the fragment is shown in dark grey. **a** and **b** show the cases of direct and reverse orientation of the fragment on the reference chromosome, respectively

**Fig. 3:**
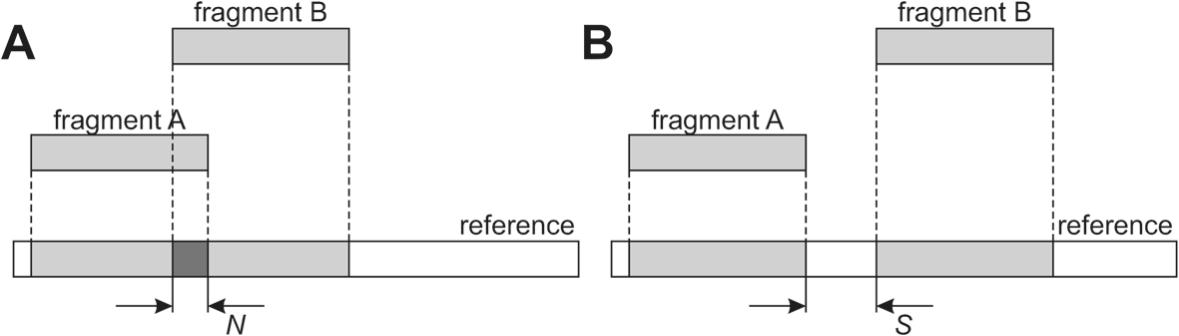
Resolving overlaps between mapped fragments. **a** shows two mapped fragments that form an overlap of *N* bp. **b** shows the fragment B shifted by (*N*+*S*) bp to resolve the overlap. *S* denotes the size of the gap inserted between the overlapping fragments and is the parameter of Chromosomer

1. From pairwise alignments, determine fragments that can be anchored to a reference according to the ratio of their first and second greatest alignment scores. If the ratio is greater than the predefined threshold, which is the algorithm parameter, then the fragment is anchored to a position corresponding to its alignment with the greatest score. Otherwise, the fragment is considered unplaced if these two alignments are located on different reference chromosomes or unlocalized if both alignments are located on the same chromosome.
2. Using fragment anchors, map the fragments to the reference chromosomes (see Fig. 2a and b). Unlocalized and unplaced fragments are excluded from the assembly.
3. Resolve overlaps between mapped fragments by inserting gaps between them (see Fig. 3a and b).
4. Produce a map describing fragment positions at a reference genome and output assembled chromosome sequences and lists of unlocalized and unplaced fragments.

Besides reference-assisted chromosome assembly, Chromosomer also offers the following options:

- transfer annotations from fragments to assembled chromosomes using a fragment map;
- visualize a reference-assisted chromosome assembly as a genome browser track containing fragment positions;
- obtain statistics on a reference-assisted chromosome assembly.

We further describe several aspects of the Chromosomer workflow: mapping fragments to reference chromosomes, transferring annotations from fragments to the assembled chromosomes and defining parameters that tune the Chromosomer assembly process. We consider all sequence coordinates to be zero-based and half-opened (that is, the first nucleotide is considered as position 0 and the last nucleotide position is equal to the sequence length).

#### Mapping fragments to reference genome

Assume we have a fragment of length *L* base pairs (bp) and an anchor between it and a reference chromosome that is formed by the alignment of the $[\overline {S}_{A}, \overline {E}_{A})$ region in the fragment and the [*S*_*A*_,*E*_*A*_) region in the reference. The $\overline {S}_{A}$ and $\overline {E}_{A}$ terms denote start and end coordinates of the alignment in the fragment and the *S*_*A*_ and *E*_*A*_ terms denote start and end coordinates of the alignment in the reference genome. We derive fragment coordinates *S*_*F*_ and *E*_*F*_ in the reference genome for two cases: a direct fragment orientation that is the same as in the reference (Fig. 2a) and an orientation that is reversed relative to the reference (Fig. 2b). Equations for *S*_*F*_ and *E*_*F*_ in the direct orientation case are:

$$S_{F} = S_{A} - \overline{S}_{A},\ E_{F} = (S_{A} - \overline{S}_{A}) + L. $$

Equations for *S*_*F*_ and *E*_*F*_ in the reversed orientation case are:

$$S_{F} = (E_{A} + \overline{S}_{A}) - L,\ E_{F} = E_{A} + \overline{S}_{A}. $$

#### Transferring annotations to assembled chromosomes

**Fig. 4:**
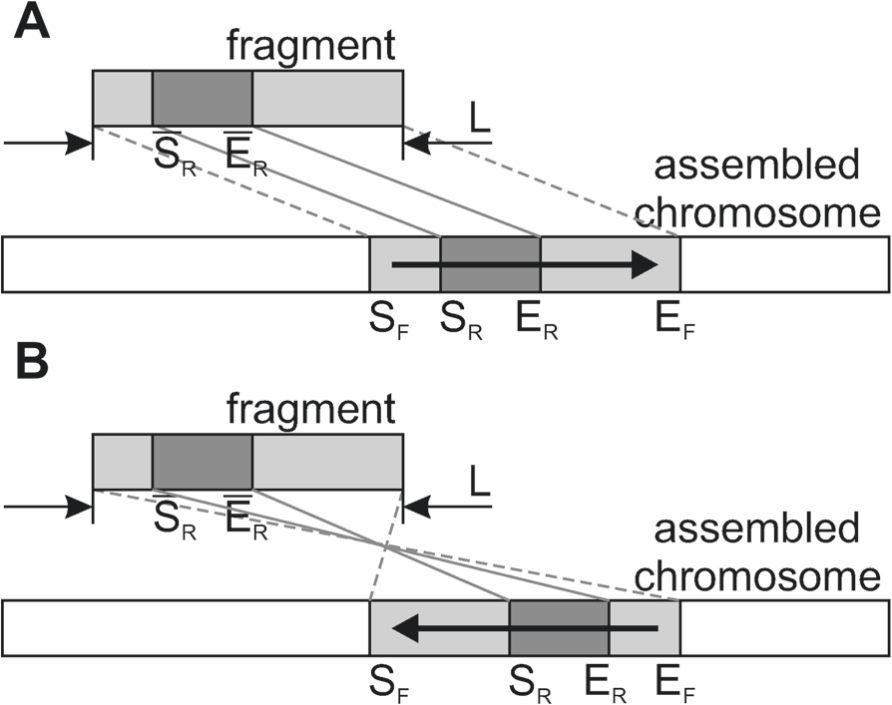
Transferring a fragment region to an assembled chromosome. The region is shown in dark grey and can represent an annotated genomic feature (e.g., a gene, a variant, a repetitive element, etc.). **a** and **b** illustrate the cases of direct and reverse fragment orientation of the fragment on the assembled chromosome, respectively

Next, assume we have a fragment of length *L* bp that is mapped to position [ *S*_*F*_,*E*_*F*_) in an assembled chromosome. We are interested in where the region $[\overline {S}_{R}, \overline {E}_{R})$ of the original fragment will be placed. Let *S*_*R*_ and *E*_*R*_ be the start and end positions of the region in the chromosome; then the following equations hold if the fragment is mapped in direct orientation (Fig. 4a):

$$S_{R} = S_{F} + \overline{S}_{R},\ E_{R} = S_{F} + \overline{E}_{R}. $$

If the region is mapped in the reverse orientation, then *S*_*R*_ and *E*_*R*_ satisfy the following equations (Fig. 4b):

$$S_{R} = E_{F} - \overline{E}_{R},\ E_{R} = E_{F} - \overline{S}_{R}. $$

#### Assembly parameters

Chromosomer introduces two parameters that influence the assembly process. The first parameter is the alignment score ratio threshold, which is used to distinguish anchored and unplaced fragments. If the score ratio of the two fragment alignments with the highest scores exceeds the threshold, then the fragment is considered anchored, otherwise it is considered unplaced and is excluded from further analysis. The alignment score ratio threshold must be a positive number greater than one.

The second parameter is the insertion size – the size of a gap which is inserted between overlapping regions (see Fig. 3b). The insertion size is recommended to be equal to or greater than the sequencing library size.

### Chromosomer assembly evaluation

To evaluate the performance of Chromosomer, we assembled the following bacterial, yeast and mammalian genomes.

1. *Escherichia coli* Sakai strain (*E. coli* K-12 strain as a reference);
2. *Saccharomyces cerevisiae* CLIB324 strain (*S. cerevisiae* S288c strain as a reference);
3. *Pantholops hodgsonii* (Tibetan antelope; *Bos taurus* as a reference);
4. *Pan troglodytes* (chimpanzee; *Homo sapiens* as a reference).

We also assembled the bacterial and yeast genomes using ABACAS and compared ABACAS-derived assemblies with Chromosomer-derived ones. Although ABACAS is not designed for assembling multichromosome genomes, we used separate ABACAS runs for each chromosome from the reference genome. The Chromosomer assembly of Tibetan antelope was compared with the RACA assembly presented in [19]. The Chromosomer-derived chimpanzee chromosomes were assessed by comparison with the GenBank assembly and by checking the coding region accuracy. LASTZ [23] was used to perform whole-genome alignments for assessing chromosomes obtained with Chromosomer.

#### *Escherichia coli* assembly

**Fig. 5:**
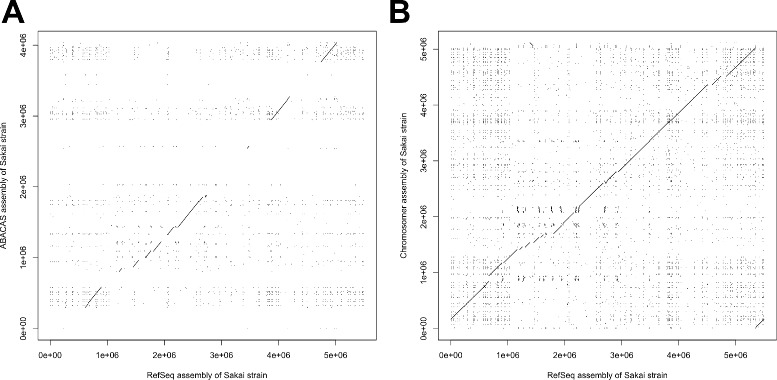
Comparison of ABACAS- and Chromosomer-produced *E. coli* Sakai strain assemblies with the RefSeq assembly. **a** and **b** show dot plots of the LASTZ alignments of the RefSeq assembly to the ABACAS and Chromosomer assemblies, respectively

The *E. coli* Sakai strain genome was assembled in two steps. First, we assembled its reads (SRA accession numbers SRR530851 and SRR587217) to scaffolds using the SPAdes assembler [24] (Additional file 1). Next, we applied Chromosomer and ABACAS to assemble the scaffolds using the *E. coli* K-12 strain genome assembly (RefSeq accession number NC_000913.3) as a reference (Additional files 2 and 3). Finally, we compared the derived assembly with the RefSeq assembly of the *E. coli* Sakai strain (RefSeq accession number NC_002695.1). The dot plots of LASTZ whole-genome alignments between the derived assemblies and the RefSeq assembly are given in Fig. 5a and b. The comparison of the assemblies is given in Table 1.

**Table 1:** Comparison of ABACAS and Chromosomer *E. coli* Sakai strain assemblies

	ABACAS	Chromosomer
Mean identity (%)	90.34	89.50
Mean length (in bp)	854.62	719.76
Mean mismatches (in bp)	21.11	22.35
Coverage (in bp)	1,999,204	5,053,547

The table shows statistics derived from LASTZ alignments of ABACAS and Chromosomer assemblies to the RefSeq *E. coli* Sakai strain assembly

#### *Saccharomyces cerevisiae* assembly

**Fig. 6:**
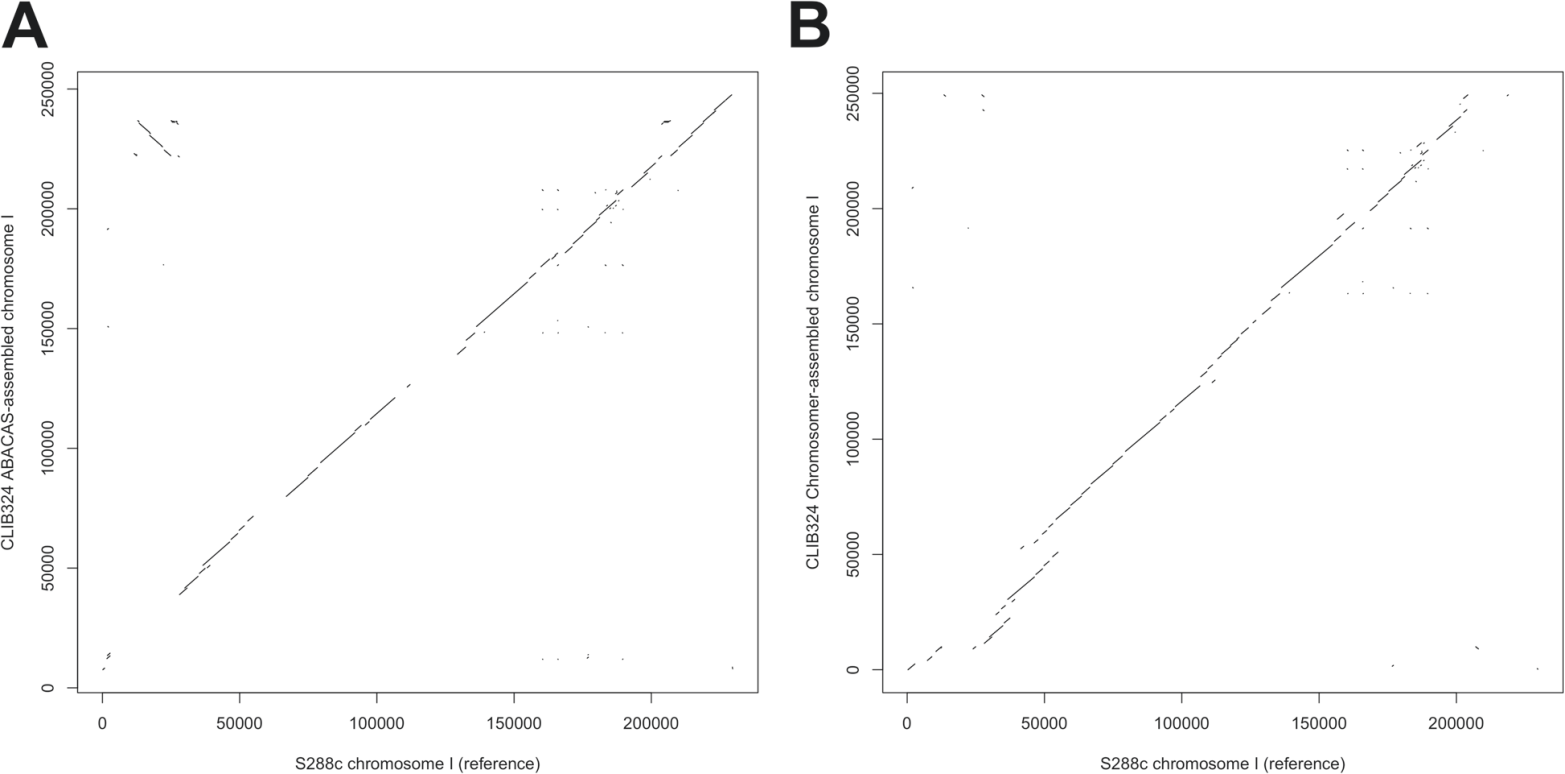
Comparison of ABACAS- and Chromosomer-produced assemblies of *S. cerevisiae* CLIB324 strain chromosome 1. The dot plots show the LASTZ alignments of the assembled *S. cerevisiae* CLIB324 chromosome 1 to the *S. cerevisiae* S288a chromosome 1 that was used as a reference for the assembly. **a** and **b** correspond to ABACAS and Chromosomer assemblies, respectively

The *S. cerevisiae* CLIB324 strain genome was assembled from its scaffolds (GenBank accession number GCA_000192495.1) using *S. cerevisiae* S288c strain genome as a reference (RefSeq accession number GCF_000146045.2). The chromosome sequences assembled by ABACAS and Chromosomer are given in Additional files 4 and 5, respectively. Dot plots comparing the LASTZ alignments of chromosome 1 between the reference genome and those from the ABACAS or Chromosomer assemblies are shown in Fig. 6a and b. The comparison of the assemblies is given in Table 2.

**Table 2:** Comparison of ABACAS and Chromosomer *S. cerevisiae* CLIB324 strain assemblies

	ABACAS	Chromosomer
Mean identity (%)	93.29	93.87
Mean length (in bp)	792.84	801.59
Mean mismatches (in bp)	14.99	11.85
Coverage (in bp)	7,920,800	9,595,323

The table shows statistics derived from LASTZ alignments of ABACAS and Chromosomer assemblies compared with the *S. cerevisiae* S288c strain assembly that was used as a reference

#### *Pantholops hodgsonii* assembly

The *P. hodgsonii* genome was assembled from its scaffolds (GenBank accession number GCA_000400835.1) using the *B. taurus* UMD3.1 assembly as a reference and the net alignments between the scaffolds and the cow chromosomes from [19]. The fragment map of the Chromosomer-derived Tibetan antelope chromosomes is given in Additional file 6.

**Fig. 7:**
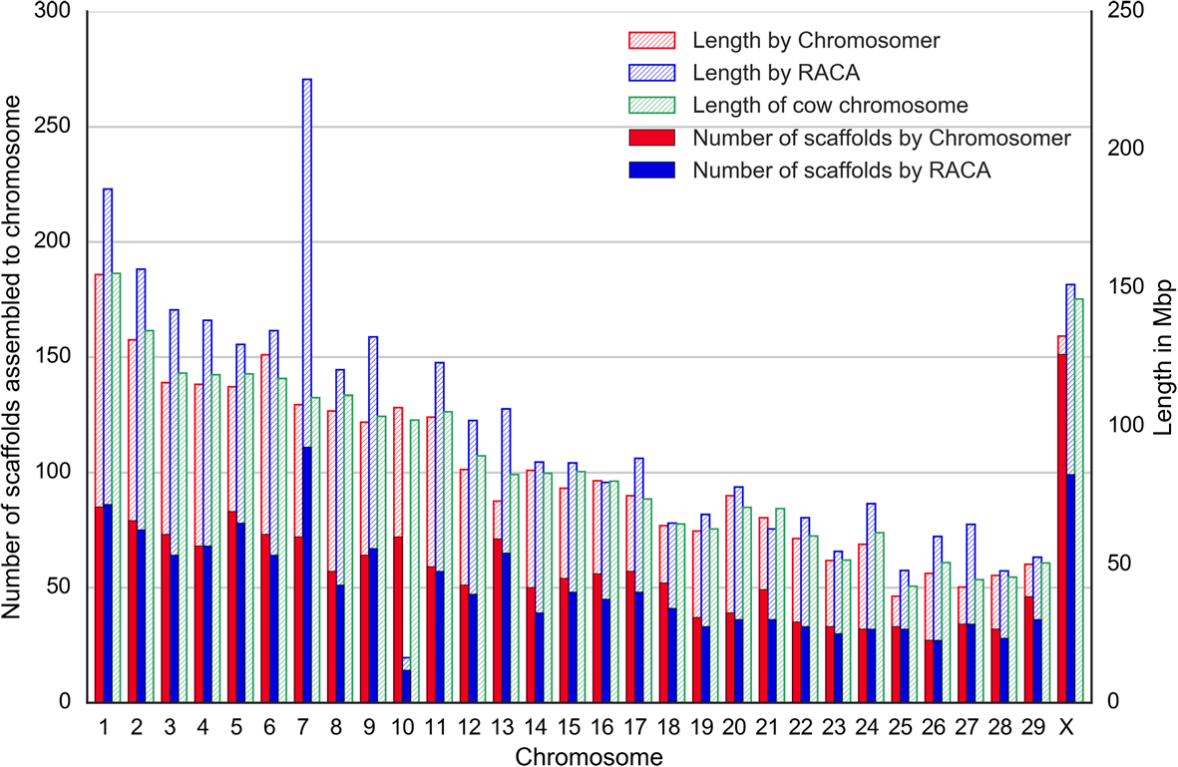
Comparison of *P. hodgsonii* predicted chromosome fragments assembled by RACA and chromosomes assembled by Chromosomer. Net alignments of the *P. hodgsonii* scaffolds to the *B. taurus* chromosomes were used in both cases

**Fig. 8:**
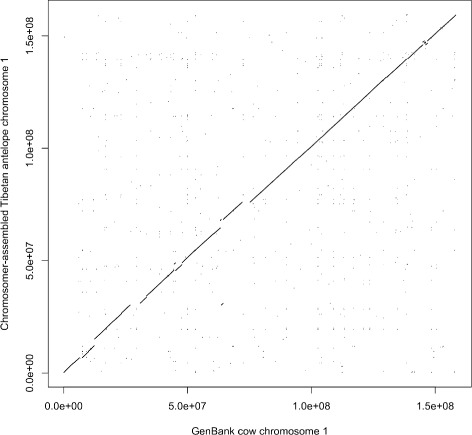
Comparison of the Chromosomer-assembled *P. hodgsonii* chromosome 1 with the cow chromosome 1. The dot plot shows the LASTZ alignments of the Chromosomer-assembled chromosome 1 to the *B. taurus* chromosome 1 that was used as a reference

We compared the Tibetan antelope chromosomes obtained by Chromosomer and the PCFs produced by RACA in Fig. 7. The comparison shows that both sets of chromosomes are similar to each other; however, RACA-derived PCFs are longer than Chromosomer-derived ones and the reference cow chromosomes. This result may be due to the difference in the Chromosomer and RACA algorithms: while RACA tends to gather as many genome fragments as possible to a larger fragment, Chromosomer determines scaffolds that have sufficient evidence for being placed on a chromosome; otherwise, Chromosomer considers a scaffold unlocalized or unplaced and does not include it in a chromosome. Thus, Chromosomer preserves the structure of the reference chromosomes, see Fig. 8.

Although the assemblies are fairly similar, two main differences can be distinguished:

1. The PCFs assembled by RACA tend to be longer than the original reference genome (cow) chromosomes.
2. RACA predicted two chromosomal translocations in the Tibetan antelope genome compared with the cow genome: the first one between chromosomes 7 and 10 and the second one between chromosomes 21 and 27. The predicted translocations led to elongation of chromosome 7 and shortening of chromosome 10; chromosomes 21 and 27 are also related in the same way but to a lesser extent (see Fig. 7). The ability to detect cross-species rearrangements is a feature of RACA that is related to its more complex assembly model and integration of paired-end reads, which Chromosomer does not use.

In addition, Chromosomer demonstrated better time performance and required fewer computational resources than RACA. It took about 1.7 hours and 1.5 GB of random access memory (RAM) for Chromosomer to produce the chromosomes from the net alignments using one CPU (central processing unit). RACA spent 55 hours and required 59 GB of RAM using three CPUs to get the result from the same net alignments. We used the SuperMicro server for the benchmark (12 Intel Xeon E5-2690 CPUs and 396 GB RAM).

#### *Pan troglodytes* assembly

**Fig. 9:**
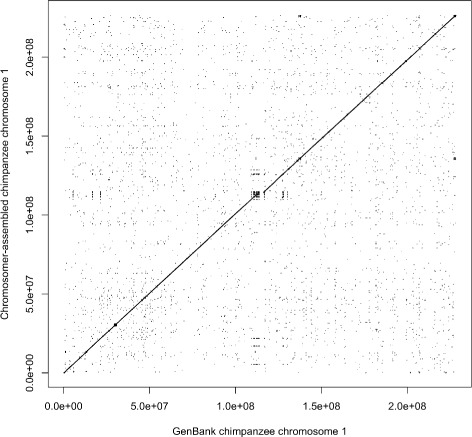
Comparison of the Chromosomer-assembled *P. troglodytes* chromosome 1 with the GenBank chromosome 1. The chromosome was assembled from the scaffolds using their net alignments to the reference genome (*H. sapiens*). The dot plot shows the LASTZ alignments of the Chromosomer-assembled chromosome 1 to its GenBank sequence

The *P. troglodytes* genome was assembled using Chromosomer from its scaffolds (GenBank assembly accession GCA_000001515.4) using the *H. sapiens* GRCh38.p2 assembly as a reference and the net alignment of the chimpanzee genome to the human genome from the UCSC Genome Browser [25, 26]. The fragment map constructed by Chromosomer is given in Additional file 7. The dot plot of the alignment of chromosome 1 assembled by Chromosomer from the scaffolds to its GenBank sequence is given in Fig. 9.

**Fig. 10:**
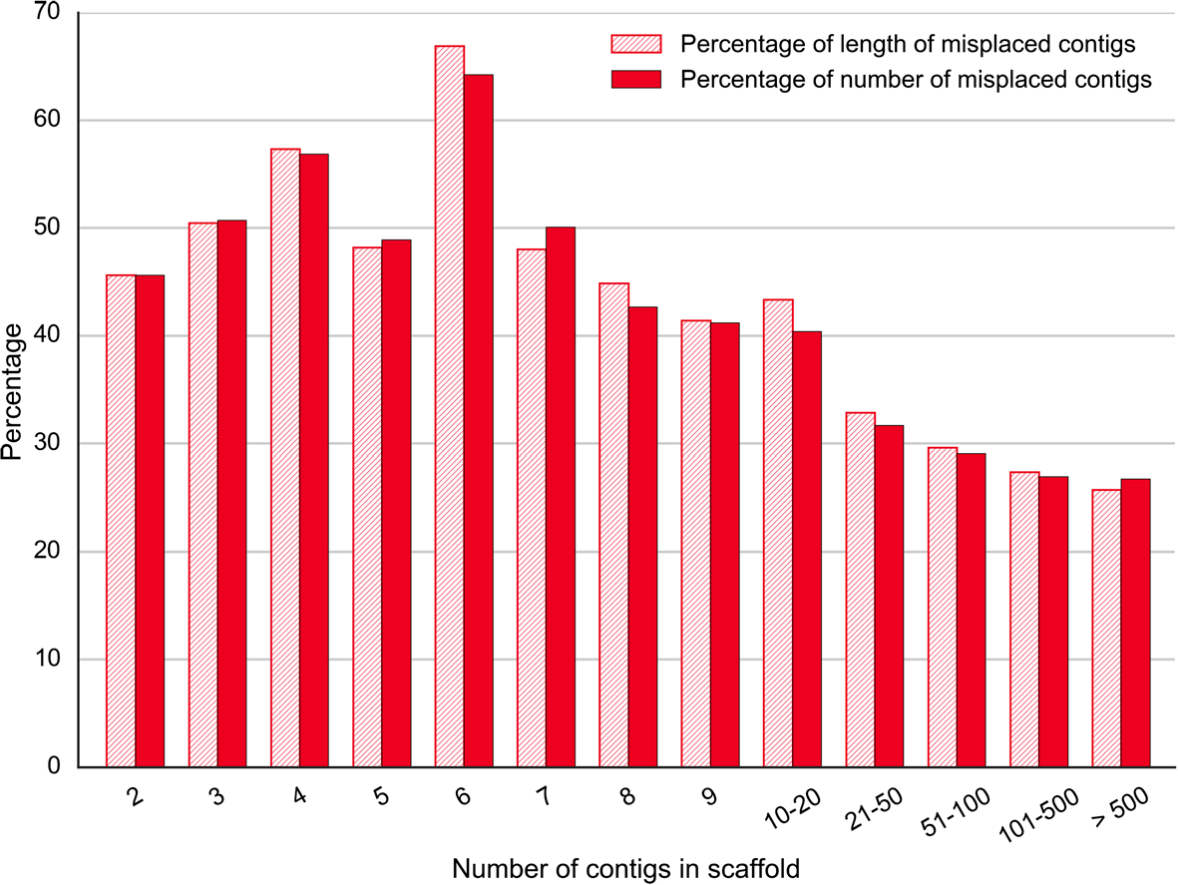
*P. troglodytes* contigs misplaced by Chromosomer within scaffolds. Only scaffolds consisting of two or more contigs were considered. A contig was considered misplaced if its neighboring contigs were different from the neighboring contigs in the GenBank assembly. For each scaffold, the percentage of its misplaced contigs and the percentage of the total misplaced contig length were calculated; the average values for all scaffolds of the specified contig number are shown

**Fig. 11:**
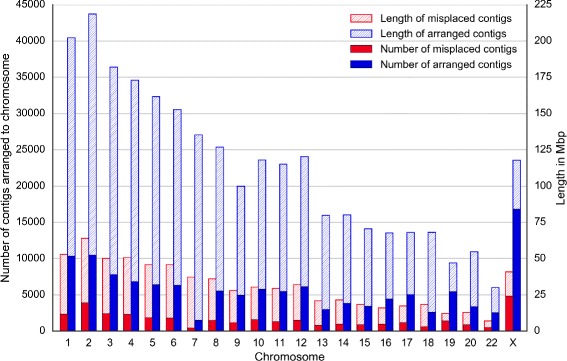
*P. troglodytes* contigs misplaced by Chromosomer. A contig was considered misplaced if its neighboring contigs were different from the neighboring contigs in the GenBank assembly

**Fig. 12:**
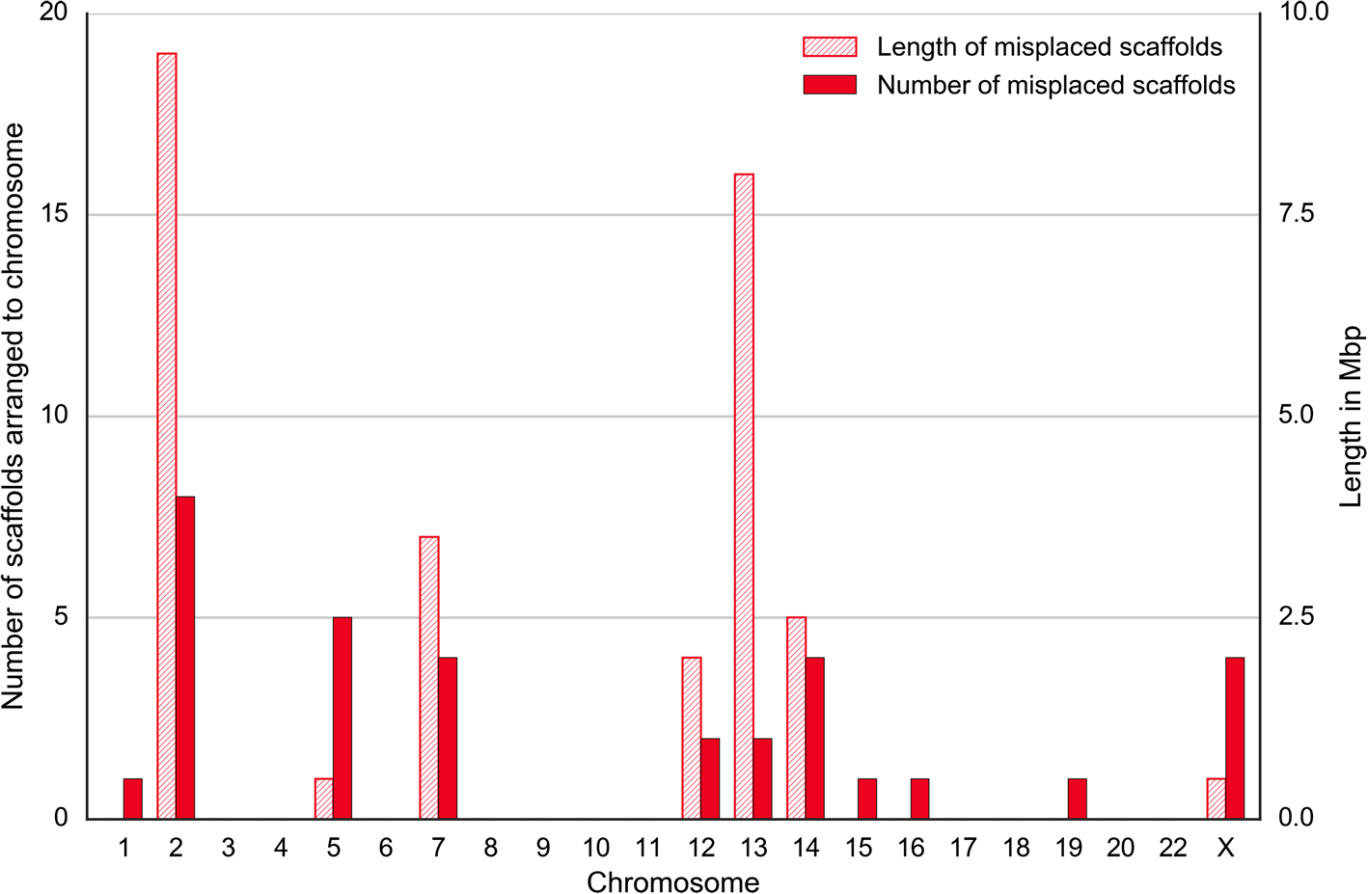
*P. troglodytes* scaffolds misplaced by Chromosomer. A scaffold was considered misplaced if its neighboring scaffolds were different from the neighboring scaffolds in the GenBank assembly. Chromosomes 1, 15, 16 and 19 contained only single misplaced scaffolds, whose lengths were 50.32 kbp, 14.23 kbp, 14.58 kbp and 1.06 kbp, respectively

**Fig. 13:**
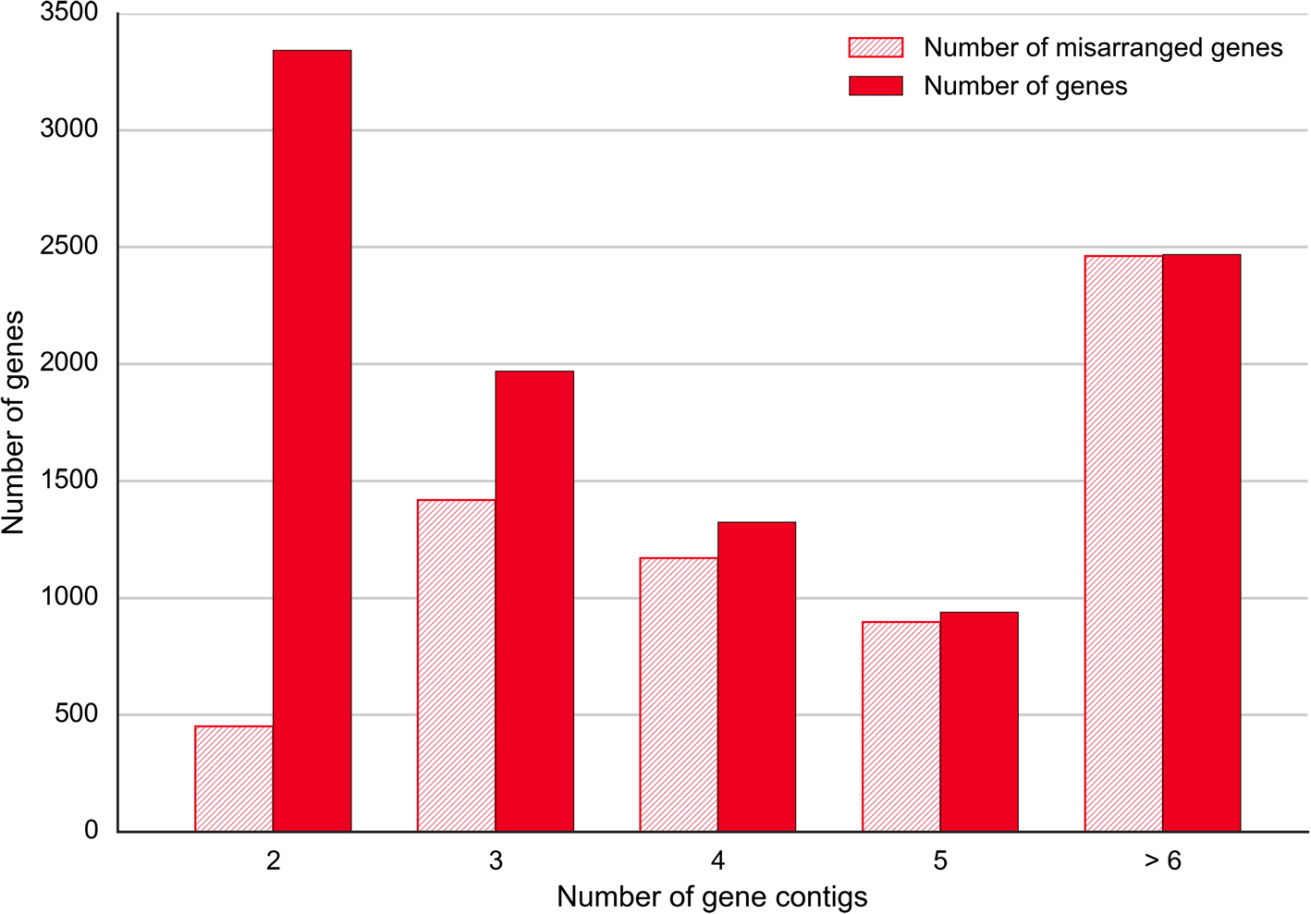
*P. troglodytes* genes on contigs misarranged by Chromosomer. Genes located on two or more contigs were considered; there were 10,041 such genes. A gene was considered misarranged if the contigs it was located on were placed in an order that differed from the GenBank assembly

**Fig. 14:**
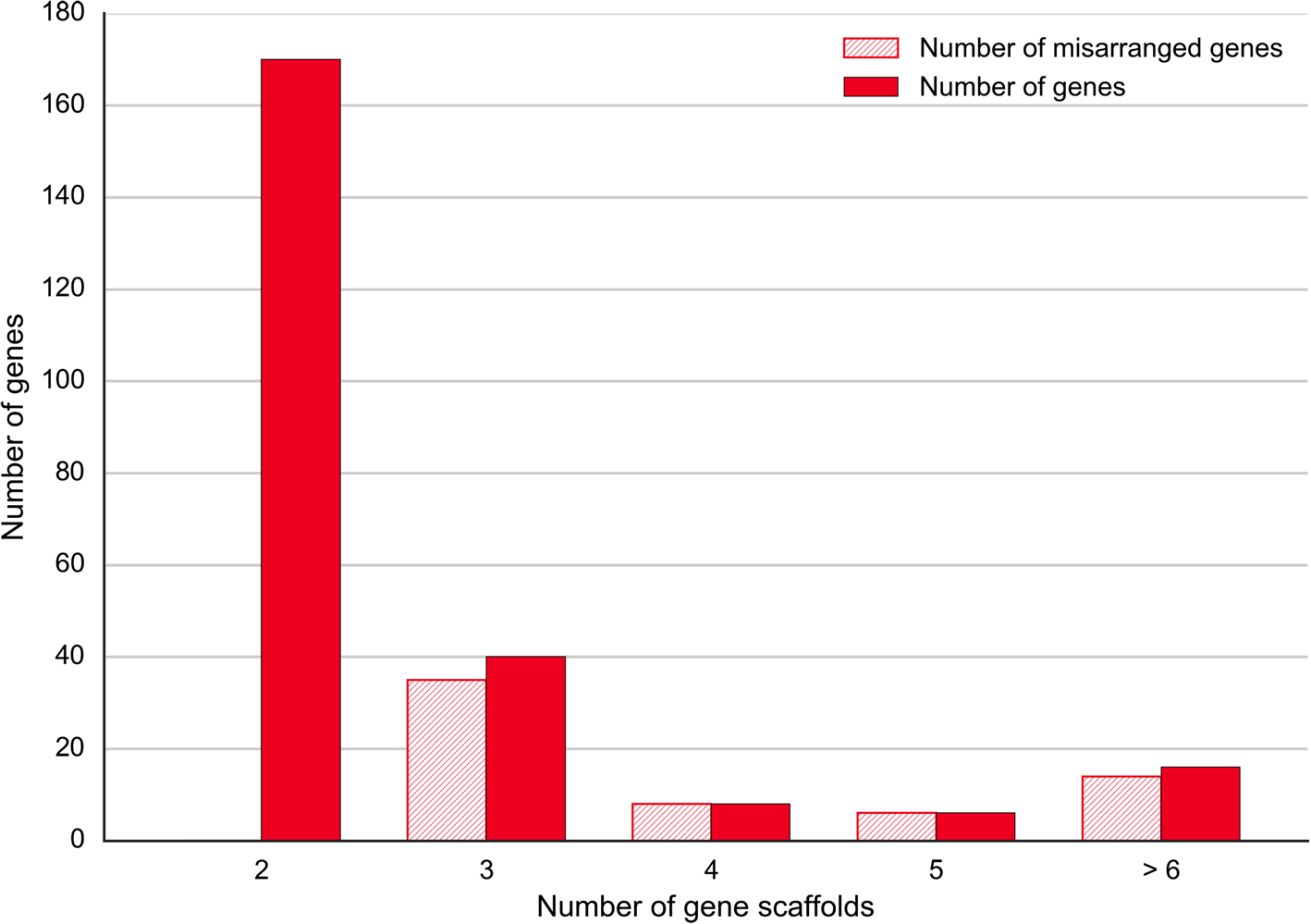
*P. troglodytes* genes on scaffolds misarranged by Chromosomer. Genes located on two or more scaffolds were considered; there were 240 such genes. A gene was considered misarranged if the scaffolds it was located on were placed in an order that differed from the GenBank assembly. Among genes located on two scaffolds, there were no misarranged genes

We assessed the obtained chimpanzee chromosomes against two criteria: adjacency of contigs and scaffolds and gene integrity. We checked the adjacency for three levels: contigs within scaffolds (Fig. 10), contigs within chromosomes (Fig. 11) and scaffolds within chromosomes (Fig. 12). The figures show that Chromosomer performs best for assembling large genomic fragments such as scaffolds and may misplace short genomic fragments like contigs for the local structure. This conclusion is supported by the gene integrity check (Figs. 13 and 14), which shows that Chromosomer might break genes located on multiple contigs.

From the examples shown above, we conclude that Chromosomer is comparable to existing reference- genome assembly tools and is able to assemble and process large genomes. Chromosomer may increase efficiency of genome annotation studies by replacing numerous genome fragments with draft chromosome assemblies.

### Availability and requirements

Chromosomer is publicly available at the Python Package Index (PyPI, https://pypi.python.org) and GitHub (https://github.com/gtamazian/chromosomer).

- **Project name:** Chromosomer
- **Project home page:**https://github.com/gtamazian/chromosomer
- **Operating systems:** Platform independent
- **Programming languages:** Python
- **Other requirements:** Python 2.7
- **License:** BSD 3-Clause License
- **Any restriction to use by non-academics:** none

## Additional files

Additional file 1
*E. coli* Sakai strain scaffolds. The FASTA file contains sequences of scaffolds assembled by SPAdes 3.6.2 from the raw reads. (FA 5488 kb)

Additional file 2 ABACAS assembly of the *E. coli* Sakai strain. The FASTA file contains the *E. coli* Sakai strain genome sequence as assembled by ABACAS from its scaffolds. (FA 4433 kb)

Additional file 3 Chromosomer assembly of the *E. coli* Sakai strain. The FASTA file contains the *E. coli* Sakai strain genome sequence as assembled by Chromosomer from its scaffolds. (FA 5068 kb)

Additional file 4 ABACAS assembly of the *S. cerevisiae* CLIB324 strain. The FASTA file contains the *S. cerevisiae* CLIB324 strain chromosome sequences as assembled by ABACAS from its scaffolds. (FA 12595 kb)

Additional file 5 Chromosomer assembly of the *S. cerevisiae* CLIB324 strain. The FASTA file contains the *S. cerevisiae* CLIB324 strain chromosome sequences as assembled by Chromosomer from its scaffolds. (FA 14028 kb)

Additional file 6 Fragment map of the Tibetan antelope genome. The fragment map of the Tibetan antelope genome chromosomes constructed by Chromosomer from net alignments of the Tibetan antelope scaffolds to the cow chromosome sequences. The file is in the Chromosomer fragment map format. (TXT 165 kb)

Additional file 7 Fragment map of the chimpanzee genome. The fragment map of the chimpanzee genome chromosomes constructed by Chromosomer from net alignments of the chimp scaffolds to the human chromosome sequences. The file is in the Chromosomer fragment map format. (TXT 263 kb)
